# Theoretical Research of a Transcritical Refrigeration System of CO_2_ Coupled with Liquid Desiccant Dehumidification Cycle Using Exergy Analysis Method

**DOI:** 10.3390/e28040436

**Published:** 2026-04-13

**Authors:** Xiao Liang, Yongbao Liu, Qiaolian Feng, Yongsheng Su, Yanfei Li

**Affiliations:** College of Power Engineering, Naval University of Engineering, Wuhan 430033, China

**Keywords:** CO_2_ transcritical refrigeration cycle, liquid desiccant dehumidification, coupled system, air conditioning, exergy analysis

## Abstract

Aiming to improve cooling and dehumidification performance in air conditioning systems and to meet the trend toward environmentally friendly refrigerants, this study proposes a coupled system that combines a CO_2_ transcritical refrigeration cycle (CTRC) with a liquid desiccant dehumidification cycle. The system takes advantage of high-grade waste heat from the exothermic side of the CTRC to drive the regenerating process of the liquid desiccant dehumidification. A cooling evaporator is adopted to cool indoor air, while another evaporator (i.e., Evaporator II) is utilized to cool the concentrated solution, improving dehumidification capacity and enabling independent control of sensible and latent heat loads. Through thermodynamic modeling and the exergy analysis model, a mathematical model of the system is developed to examine how key parameters (such discharge pressure and the CO_2_ mass flow rate ratio in Evaporator II (*λ*)) affect performance and to analyze exergy loss features. Results show that the system’s coefficient of performance (COP) and dehumidification coefficient of performance (COP_deh_) initially rise and then fall with increasing CTRC discharge pressure, achieving an optimal pressure of around 10,500 kPa (COP up to 4.32) under a specific working condition, surpassing those of standalone CTRC systems. Properly increasing *λ* enhances dehumidification capacity and energy efficiency, with a low specific dehumidification energy (SDE) of 0.2033 kWh/kg, indicating high economic efficiency. Most exergy losses occur in the CO_2_-solution heat exchanger and dehumidifier (over 60% of total losses). The system’s maximum exergy efficiency reaches 12.4%, leaving room for further improvements. This coupled system offers an efficient, eco-friendly way for air conditioning in high-humidity environments, combining cooling and dehumidification with the potential for energy recovery.

## 1. Introduction

### 1.1. CO_2_ Transcritical Cycle

As traditional HFCs are gradually phased out under the Montreal Protocol, refrigerant replacement technologies become a key focus in air conditioning. Natural refrigerants have regained interest among researchers worldwide due to their low GWP. For example, CO_2_, which has a GWP of 1, was once widely used as a natural refrigerant and remains in use in vehicle air conditioning nowadays. Its nontoxicity, nonflammability, and chemical inactivity also make CO_2_ a competitive replacement for other refrigerants.

A refrigeration system using CO_2_ typically operates across a wide pressure range due to its thermophysical properties. CO_2_ on the exothermic side is often supercritical, with temperatures above 31 °C, leading to the so-called CO_2_ transcritical refrigeration cycle (CTRC). The temperature glide in the exothermic process makes the transcritical refrigeration cycle fundamentally different from the traditional vapor compression refrigeration cycle (VCRC). Due to the temperature glide, deviations occur between the CTRC and the ideal reversed Carnot cycle, resulting in lower efficiency. However, CTRC consistently demonstrates strong development prospects owing to its inherent advantages. The compressor achieves high efficiency because the actual pressure ratio of the CTRC is relatively low, even though the working pressure can reach 10 MPa. The excellent heat-transfer performance and thermophysical properties of supercritical CO_2_ contribute to improved heat-exchanger efficiency in CTRC, further enhancing its overall performance. As a result, researchers have implemented various strategies in recent years to address CTRC’s performance limitations. Some efforts focus on recovering or fully utilizing the high-grade thermal energy released by the exothermic CTRC process.

The most common approach is to treat CTRC as a heat pump water heater, matching the water’s temperature increase to the supercritical CO_2_’s temperature glide during the exothermic process, analogous to a specific type of Lorenz cycle. In the 1990s, Lorentzen and Pettersen conducted the first theoretical and experimental studies on the application of CTRC to heat pump water heaters [1]. A CTRC heat pump water heater prototype and its experimental setup were introduced in 1996 at the SINTEF research institute, with a power of 50 kW [2]. The experimental results showed that hot water could be heated from 9 to 50 °C at an evaporating temperature of 0 °C, with a COP reaching up to 4.3. In previous studies on CTRC heat pump water heaters, it is generally believed that hot water can be heated to higher temperatures than in traditional heat pump water heaters [3].

Amid the growing global focus on CO_2_ refrigeration technology, many scholars worldwide have recently conducted extensive research on process design optimization, performance improvement, and the promotion of key devices for CTRC. Due to the large pressure difference in CTRC, irreversible losses are mainly located in the throttling process. Therefore, to utilize the pressure energy of high-pressure supercritical CO_2_, introducing ejectors and expanders into the cycle is a relatively common method [4]. Various types of expanders, including screw, reciprocating piston, and turbo, have been shown in previous studies to improve CTRC performance using both theoretical and experimental methods effectively [5,6,7]. As a device with no moving parts, the ejector, when integrated into the CTRC, can effectively recover the pressure energy of high-pressure supercritical CO_2_. The CTRC incorporating an ejector can improve system performance by decreasing compression power, a feature confirmed by many researchers [8,9,10]. Furthermore, based on the above studies, there are additional methods—such as incorporating devices like internal heat exchangers and thermoelectric coolers—to achieve further subcooling, with the ultimate goal of enhancing the system performance of CTRC [11,12,13].

Besides improving cycle performance, many studies focus on combining the CTRC with other systems to achieve refrigeration while effectively utilizing high-temperature discharge heat. It is common practice to combine the CTRC with both refrigeration loads and heating circuits. By virtue of its high-temperature discharge and variable-temperature heating capabilities, it can effectively achieve hot water heating, thus improving the overall energy utilization efficiency of the system [14]. Other examples include integrating the discharge side of the CTRC for vehicle air conditioning with a battery preheating system and combining the discharge side of the CTRC for food refrigeration air conditioning with a food desiccant regeneration system. A wide variety of auxiliary systems are integrated with CTRC to utilize discharge heat, all aiming to improve overall system energy efficiency [15,16].

### 1.2. Liquid Desiccant Dehumidification Cycle Coupled with Various Refrigeration Cycles

In the field of air conditioning, to better achieve the two air-handling processes of cooling and dehumidification, scholars have begun to explore combining the liquid desiccant cycle with various refrigeration cycles used in air conditioning. Combining the vapor compression refrigeration cycle with the liquid desiccant cycle enables the former’s condensation heat to drive liquid desiccant regeneration [17,18]. In the early hybrid system proposed by Dai [17], the condensation heat of the vapor compression system was not directly recovered for solution regeneration, and the regenerator was driven by external low-grade energy such as solar or waste heat. But outlet air of the condenser with relatively high temperature has been utilized to preheat the diluted solution before the regeneration process. In their study, system COP at an ideal working condition can reach nearly 6.5 through theoretical investigation. She [18] innovatively combined the vapor compression cycle with the liquid desiccant dehumidification cycle for refrigerant subcooling, which promotes the systematic performance in a special aspect. Park [19] proposed an evaporative cooling-assisted internally cooled liquid desiccant dehumidifier to promote the liquid desiccant dehumidification system performance driven by the vapor compression refrigeration cycle. Su [20] coupled the absorption refrigeration cycle with the liquid desiccant cycle, using the heat released during the cooling and condensation of the refrigerant vapor generated by the high-temperature generator to drive desiccant regeneration, intending to improve system performance.

In addition, many scholars have integrated heat pumps with liquid desiccant cycles for dehumidification, utilizing the exothermic side of the heat pump to heat the dilute solution and the endothermic side to cool the concentrated solution, thereby achieving deep dehumidification [21,22,23]. Owing to sufficient heating and cooling of the solution, some of their results indicates a COP up to 7.2 [23]. The core of all the aforementioned coupled systems lies in utilizing waste heat to drive the liquid desiccant cycle. Furthermore, existing research results indicate that the liquid desiccant dehumidification effect is directly related to the grade of the driving waste heat. But the phenomenon also brings another problem regarding how to ensure sufficient cooling of the concentrated solution. Moreover, existing studies on various coupled systems mostly focus on utilizing the condensing heat to drive desiccant regeneration, but do not mention the independent control of sensible and latent heat loads, lacking systematic optimization of the flow distribution of dual evaporators.

### 1.3. Aim of the Present Study

Based on the previous research mentioned above, this paper proposes a coupled system integrating the CTRC and the liquid desiccant dehumidification cycle to achieve controllable proportions of sensible and latent heat loads by means of dual evaporators. This design is intended to meet the requirements of various cooling and dehumidification scenarios, particularly for air conditioning in high latent heat-load environments or deep-dehumidification situations, while remaining energy-efficient. For scenarios such as environmental dehumidification in cabins with marine electronic equipment, dehumidification in electronic device manufacturing processes, and humidity control in data centers, their demand for large dehumidification loads well matches the coupled dehumidification system proposed in this study. Otherwise, the proposed coupled system owns a unique characteristic which is totally environmentally friendly—only CO_2_ and aqueous solution have been applied. Using theoretical analysis, this study conducts thermodynamic modeling of the coupled system. It analyzes the relationships between system performance-characterizing parameters (e.g., COP and exergy efficiency) and working conditions. In combination with exergy models for the desiccant solution and moist air, the features of exergy loss in the coupled system are analyzed and summarized to provide theoretical support for subsequent experimental research and further process optimization.

## 2. System Description

**Figure 1:**
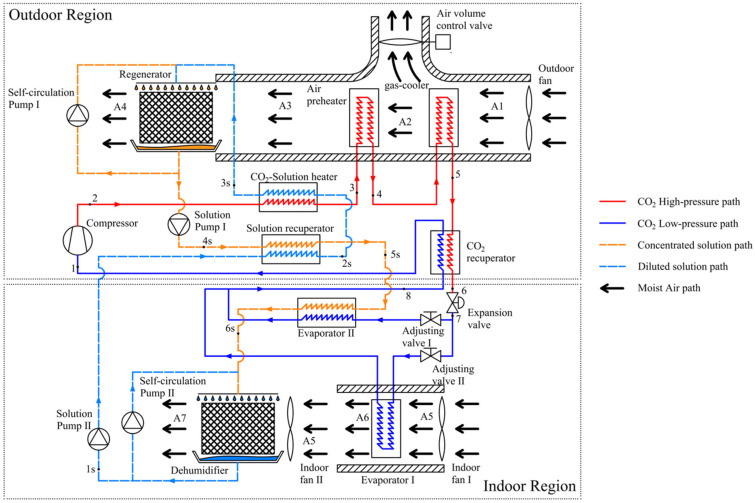
Schematic diagram of the coupled system.

The current study introduces a coupled system that fully takes advantage of the heat generated during the exothermic process of CTRC to drive a liquid desiccant dehumidification cycle. Figure 1 illustrates the structure of the coupled system, with each pathway marked by a different color.

The diagram illustrates that an air preheater and a CO_2_-solution heat exchanger are positioned on the exothermic side of the CTRC, operating in a countercurrent configuration, utilizing high-grade thermal energy from the supercritical CO_2_ cooling process. The following sections provide details of the two cycles and the functions of each component.

### 2.1. Flow Paths of CTRC and Its Components

Typically, CTRC for air-conditioning comprises four primary processes: isothermal compression, isobaric exothermic and endothermic processes, and throttling. Unlike traditional VCRC, the isobaric exothermic process for CTRC occurs at a supercritical state and is not isothermal. The discharge temperature of the compressor is often above 80 °C, and the non-isothermal heat-release process enables supercritical CO_2_ to serve as an excellent high-grade thermal source. Due to this characteristic of CTRC, the thermal energy released by supercritical CO_2_ is utilized to heat the diluted solution and moist air for the regeneration process in a countercurrent configuration in the present study.

From Figure 1, it can be seen that vaporized CO_2_ (point 1) is first sucked into the compressor and then compressed to discharge pressure with high temperature (point 2). CO_2_ at high temperature will flow through the CO_2_-solution heat exchanger, transferring heat to the solution on the other side. Due to the temperature glide, CO_2_ at the outlet of the CO_2_-solution heat exchanger (point 3) will experience a large decrease in temperature. Flowing air will absorb heat from CO_2_ at a relatively high temperature through a tube-fin heat exchanger, regarded as an air preheater. Furthermore, CO_2_ (point 4) passes through the gas-cooler, a tube-fin heat exchanger, which cools it to near-ambient temperature (point 5). At the end of the high-pressure side of the CTRC, CO_2_ receives additional cooling from a CO_2_-recuperator (point 6) before throttling. Two-phase CO_2_ obtains cooling capacity (point 7) through throttling. It then flows past evaporators I and II respectively, providing cooling that reduces the temperature of the indoor air and the concentrated solutions through evaporation. Fully vaporized CO_2_ (point 8) enters the CO_2_-recuperator to gain a superheated degree (point 1) and prepares for compression. Thus, the CTRC of the coupled system is completed. Axial fans drive heat-exchange processes in tube-fin heat exchangers.

**Figure 2:**
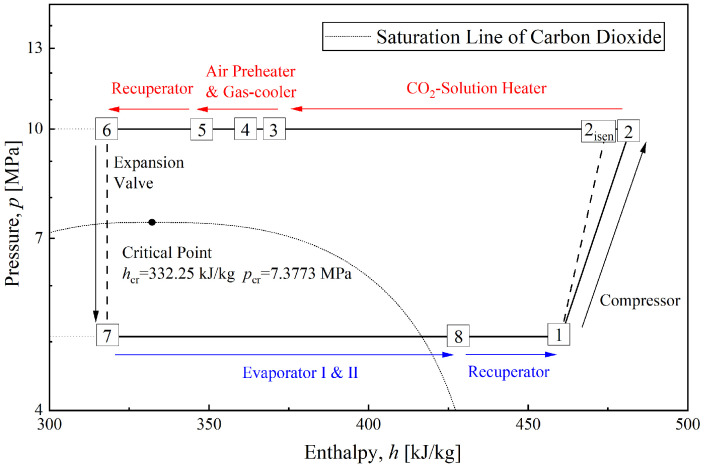
*p*-*h* diagram for a typical CTRC in the coupled system.

**Figure 3:**
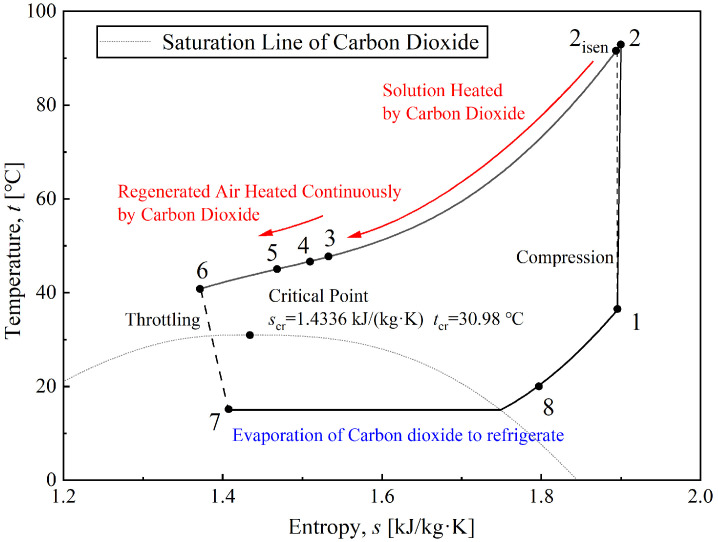
*T*-*s* diagram for a typical CTRC in the coupled system.

To better understand the CTRC circulation process, Figure 2 and Figure 3 illustrate a typical CTRC in the coupled system shown in Figure 1 using *p*-*h* and *T*-*s* diagrams. The temperature glide of the CTRC exothermic process is clearly evident in the diagram. Hence, the use of high-grade thermal energy from supercritical CO_2_ is similar to a step-type process. The high-temperature portion of the exothermic process heats the diluted solution in the liquid desiccant dehumidification cycle, whereas the lower-temperature portion preheats moist air. Moreover, part of the CO_2_ with cooling capacity is diverted to another path (i.e., evaporator II), thereby cooling the concentrated solution and increasing its dehumidification capacity. Those connections between the two cycles are the core factor for the coupled system. The detailed principles of these measures will be introduced in the following sections.

### 2.2. Liquid Desiccant Selection

**Figure 4:**
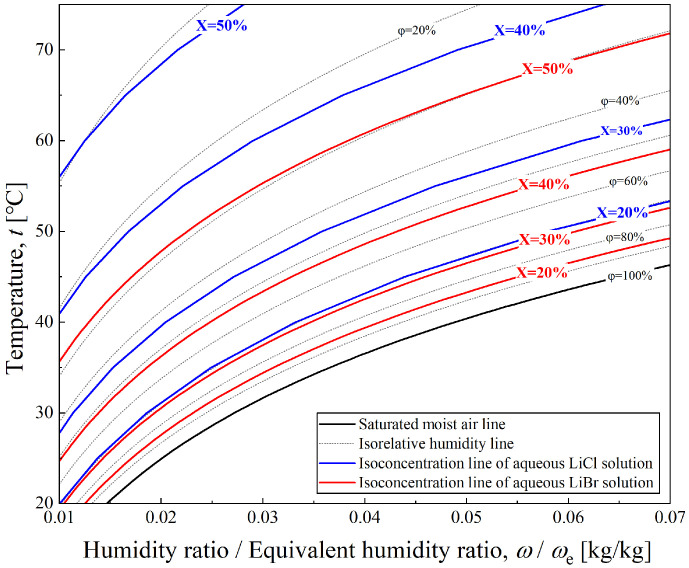
Equivalent humidity ratio comparison between aqueous LiBr and aqueous LiCl solution.

The properties of a specific type of liquid desiccant (i.e., the solution) have a decisive effect on the performance of dehumidification cycles. A significant difference in boiling point between the solute and solvent is a prerequisite for selecting a desiccant solution in liquid desiccant dehumidification. Aqueous LiBr (lithium bromide) and aqueous LiCl (lithium chloride) solutions are currently the two most widely used desiccant working pairs. Research on their physical and chemical properties, as well as their performance in practical dehumidification systems, has been relatively comprehensive. According to the research findings of Liu [24], the physical properties of aqueous LiBr and LiCl solutions are relatively similar, and their performance differences under identical operating conditions are minor. However, LiCl requires a lower concentration to achieve a given saturated vapor pressure than LiBr, as illustrated in Figure 4. Therefore, despite the higher cost of LiCl, it exhibits better overall economic efficiency in liquid desiccant dehumidification systems. Based on the factors mentioned above, the liquid desiccant dehumidification cycle in this study employs an aqueous LiCl solution as the liquid desiccant. The thermodynamic model includes relevant properties, such as saturated vapor pressure and specific enthalpy.

### 2.3. Flow Paths of Liquid Desiccant Dehumidification Cycle and Its Components

**Figure 5:**
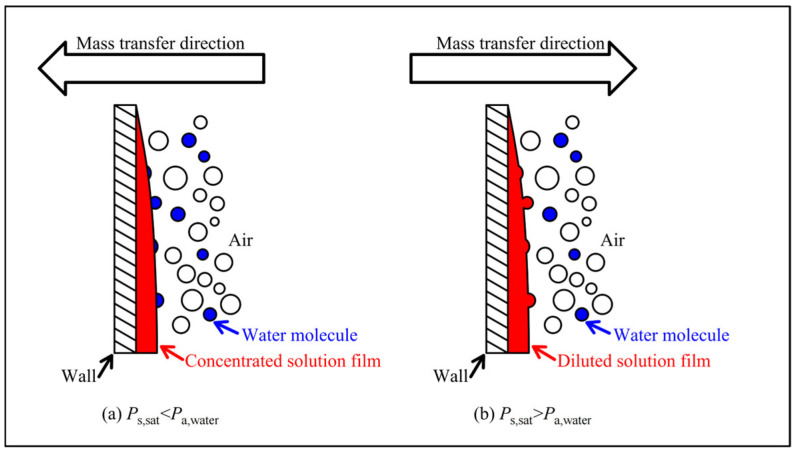
Mass transfer principle during dehumidification and regenerating process.

The liquid desiccant dehumidification cycle, as shown in Figure 1, is similar to conventional cycles. Before describing the liquid desiccant dehumidification cycle, a brief introduction to the dehumidification and regeneration modules is provided. It is commonly acknowledged that the pressure difference between the saturated pressure of solution and the partial water pressure of moist air is the core driver of heat and mass transfer in the dehumidification and regenerating processes, as shown in Figure 5. When the saturated pressure of the solution is lower than the partial water pressure of moist air, the pressure difference forces water molecules to be absorbed by the solution, which is the so-called dehumidification process. Thus, the design principle of the dehumidification cycle focuses on increasing the pressure difference described above. Dehumidification and regeneration modules are devices in which mass transfer occurs. As shown in Figure 1, both modules consist of a sprayer, a structured packing, a liquid pan, two axial fans, and a self-circulation cycle with a spray pump. The self-circulation cycle is designed to ensure comprehensive mass transfer throughout the system.

**Figure 6:**
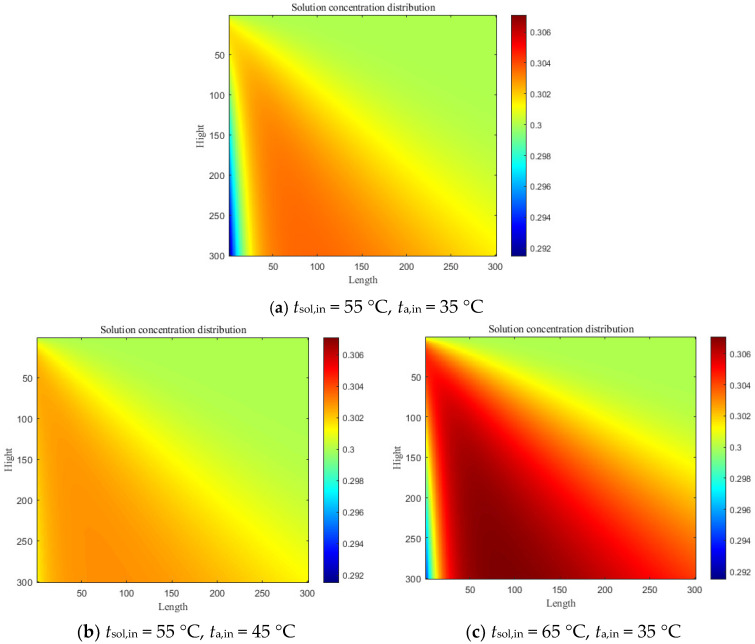
Solution concentration distribution under various boundary conditions by the finite element method.

The temperatures of the regenerating moist air and dilute solution significantly influence the intensity of the regenerative mass-transfer process. This is evident from the above descriptions of mass-transfer driving forces for processes such as dehumidification and regeneration, as well as from the authors’ preliminary finite-element analysis (proposed by Liu [25]) of the cross-flow regenerator module (moist air flows from the left while the diluted solution flows from the top). Figure 6 shows the solution concentration distributions under conditions of varying regenerating moist-air and regenerating dilute-solution temperatures. As shown in Figure 6a,b, as the temperature of the regenerating moist air increases, the concentration distribution of the dilute solution at the air inlet becomes more uniform. This is because at the moist-air inlet, the heat-transfer effect of the moist air on the high-temperature concentrated solution is significant; once the dilute solution’s temperature drops sharply in the local inlet region, it loses its regeneration capacity. Furthermore, Figure 6a,c show that increasing the temperature of the dilute solution (i.e., enhancing the mass-transfer driving force for regeneration) effectively increases the concentration of the regenerating dilute solution. Therefore, the coupled system proposed in this study is equipped with a CO_2_-solution heat exchanger on the exothermic side of the CO_2_ transcritical cycle, leveraging the high discharge temperature of the CO_2_ compressor to enhance the concentration of the solution at the outlet of regenerator. A similar relationship exists between the temperature of the concentrated desiccant solution and the intensity of dehumidification mass transfer on the dehumidifier side; therefore, Evaporator II is configured to cool the concentrated solution.

The concentrated solution at the regenerator module outlet (point 4 s) is first pumped to the solution recuperator, where it is cooled to a lower temperature (point 5 s) and a lower saturated pressure. To achieve sufficient dehumidification, a portion of the CO_2_ with cooling capacity (point 7) will cool the concentrated solution in evaporator II, which is located downstream of the solution recuperator, thereby reducing the concentrated solution temperature to the final temperature (point 6 s). At this point, the concentrated solution acquires dehumidification capability. The concentrated solution at the cooler outlet will be prepared for dehumidification and enter the dehumidification module. After the module’s complete dehumidification, the diluted solution, which absorbs water from moist air (point 1 s), is then pumped back into the solution recuperator. Heat recovery from the solution recuperator slightly increases the diluted solution temperature (point 2 s); however, the liquid desiccant dehumidification cycle primarily relies on heat transfer in the CO_2_-solution heat exchanger. The diluted solution will be heated to a very high temperature (point 3 s) and prepared for regeneration. For the regenerating process, the procedure is the same as that for dehumidification, but the mass-transfer direction is reversed. Then, all the paths of the liquid desiccant dehumidification cycle are completed.

**Figure 7:**
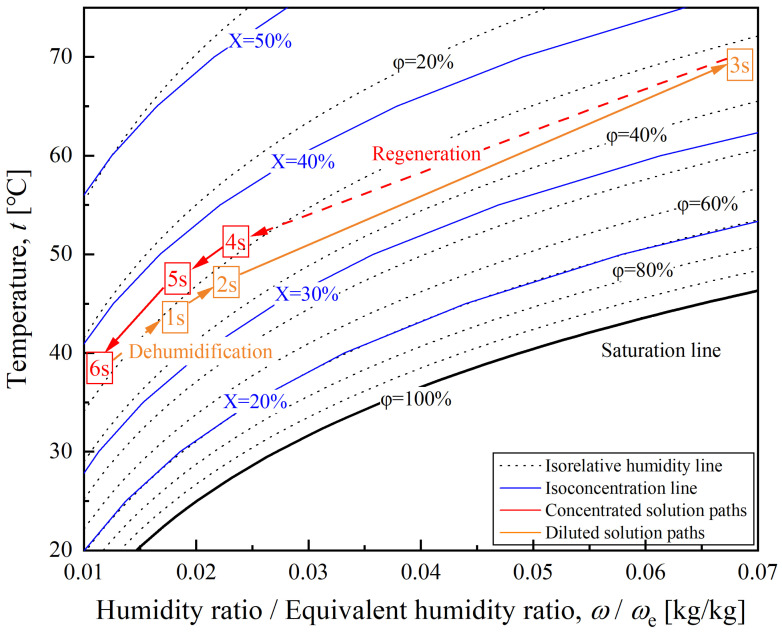
A schematic *T*-*ω* diagram of the presented liquid desiccant dehumidification cycle.

Similarly, to better understand the liquid desiccant dehumidification cycle, the state of each point on the *T*-*ω* diagram is shown in Figure 7. The equivalent humidity ratio, *ω*_e_, is a state parameter of the solution that quantifies its dehumidifying capacity. The physical meaning of *ω*_e_ is the humidity ratio of moist air that is in vapor–liquid equilibrium with the solution. It can be calculated using Equation (1).

(1) $$\omega_{e}=0.622\frac{p_{\mathrm{sat},\mathrm{sol}}}{p_{\mathrm{at}}-p_{\mathrm{sat},\mathrm{sol}}}$$

From the descriptions above, it can be concluded that the CO_2_-solution heat exchanger, air preheater, and evaporator II are the critical components of the coupled system. Both aim to promote mass transfer during regeneration and dehumidification. The CO_2_-solution heat exchanger is designed to increase the diluted solution temperature and its corresponding saturated pressure. The air preheater is used to reduce the relative humidity of moist air through isohume heating, enhancing moisture absorption. Evaporator II helps reduce the temperature of the concentrated solution and the saturated pressure, thereby improving the mass-transfer driving force. Therefore, in the external environment region, a moist-air path is configured to pass directly over the gas-cooler and air preheater, thereby minimizing the air’s relative humidity. The air-volume control valve is set to adjust the air mass flux to the downstream heating devices.

In the air-conditioning system, the dehumidification module and Evaporator I operate independently, each serving two moist-air paths. From the coupled system, it can be learnt that the distribution of the CO_2_ mass flow rate between Evaporator I and Evaporator II is a key factor affecting the system performance. The former directly determines the cooling capacity of the indoor air. At the same time, the latter determines the extent of temperature reduction in the concentrated solution, thereby enhancing the dehumidifier’s dehumidification performance.

## 3. Methodology

### 3.1. Systematic Modeling

To obtain comprehensive performance characteristics, especially the exergy performance of the coupled system under various conditions, theoretical methods are based on the governing equations of each component and process.

#### 3.1.1. Governing Equations of CTRC

Initially, the status of the CTRC should be determined along with the energy equilibrium of the corresponding parts. The necessary thermophysical properties of CO_2_ are available via the NIST REFPROP program.

##### Compressor

The displacement of a specific type of compressor is assumed constant. Thus, the refrigerant mass flow rate of CTRC can be calculated by Equation (2) with the particular volume of CO_2_ at the suction pressure (point 1).

(2) $${\dot{m}}_{r}=\frac{V}{v_{1}}$$

The compressor discharge state is determined using the isentropic process and isentropic efficiency, as given in the following Equations (3) and (4).

(3) $$s_{1}=s_{2s}$$

(4) $$\eta_{\mathrm{isen}}=\frac{h_{2,\mathrm{isen}}-h_{1}}{h_{2}-h_{1}}$$

Finally, the compressor power can be derived from Equation (5).

(5) $$W=\left(h_{2}-h_{1}\right)\cdot {\dot{m}}_{r}$$

##### CO_2_-Solution Heat Exchanger

The CO_2_-solution heat exchanger transfers heat between supercritical CO_2_ and the concentrated solution. Energy equilibrium is the basic principle for heat exchange devices of the whole coupled system, in the form of Equation (6) in the case of the CO_2_-solution heat exchanger.

(6) $$Q_{{\mathrm{HX},\mathrm{CO}}_{2}\mathrm{-sol}}={\dot{m}}_{r}\cdot \left(h_{2}-h_{3}\right)={\dot{m}}_{\mathrm{sol}}\cdot \left(h_{3s}-h_{2s}\right)$$

##### Air Preheater & Gas-Cooler

Similar to Equation (6), the energy equilibrium between the air preheater and the gas-cooler is between CO_2_ and moist air, with the governing equations as follows.

(7) $$Q_{\mathrm{HX},\mathrm{preheater}}={\dot{m}}_{r}\cdot \left(h_{3}-h_{4}\right)={\dot{m}}_{a,A2\text{-}A3}\cdot \left(h_{A3}-h_{A2}\right)$$

(8) $$Q_{\mathrm{HX},\mathrm{gas-cooler}}={\dot{m}}_{r}\cdot \left(h_{4}-h_{5}\right)={\dot{m}}_{a,A1\text{-}A2}\cdot \left(h_{A2}-h_{A1}\right)$$

##### Recuperator of CO_2_

The present device is a common component of CTRC, providing an additional degree of subcooling for CO_2_ at the inlet of the throttling valve. Thus, the energy equilibrium can be described by Equation (9).

(9) $$Q_{{\mathrm{Recuperator},\mathrm{CO}}_{2}}={\dot{m}}_{r}\cdot \left(h_{5}-h_{6}\right)={\dot{m}}_{r}\cdot \left(h_{1}-h_{8}\right)$$

##### Evaporator

As a critical component of the CTRC, the evaporators are responsible for air conditioning and for cooling concentrated solutions in the coupled system. The mass flow rates of refrigerant CO_2_ for the two evaporators are set according to a specified proportion, as shown in Equation (10). From both the refrigerant and moist-air sides, the cooling capacities of the two evaporators can be determined from Equations (11) and (12).

(10) $${\dot{m}}_{r}={\dot{m}}_{r,I}\cdot \left(1-\lambda \right)+{\dot{m}}_{r,\mathrm{II}}\cdot \lambda$$

(11) $$Q_{e,I}={\dot{m}}_{r,I}\cdot \left(h_{5}-h_{6}\right)={\dot{m}}_{a,A5\text{-}A6}\cdot \left(h_{A5}-h_{A6}\right)$$

(12) $$Q_{e,\mathrm{II}}={\dot{m}}_{r,\mathrm{II}}\cdot \left(h_{5}-h_{6}\right)={\dot{m}}_{\mathrm{sol},\mathrm{con}}\cdot \left(h_{5s}-h_{6s}\right)$$

#### 3.1.2. Governing Equations of Liquid Desiccant Dehumidification Cycle

Unlike CTRC, solution concentration is an essential parameter in mass-transfer calculations during dehumidification and regeneration. In the present model, thermophysical properties, such as the saturated pressure and specific enthalpy of LiCl solution, are determined based on the studies by Conde [26] and Chaudhari [27]. Detailed correlations are presented in Equations (13)–(22), along with their coefficients in Table 1 and Table 2.

(13) $$P_{\mathrm{sat},\mathrm{sol}}\left(X,t\right)=P_{{\mathrm{sat},H}_{2}O}(t)\cdot \pi_{25}\cdot f\left(X,\theta \right)$$

(14) $$f\left(X,\theta \right)=A+B\cdot \theta$$

(15) $$A=2-{\left[1+{\left(\frac{X}{\pi_{0}}\right)}^{\pi_{1}}\right]}^{\pi_{2}}$$

(16) $$B={\left[1+{\left(\frac{X}{\pi_{3}}\right)}^{\pi_{4}}\right]}^{\pi_{5}}-1$$

(17) $$\theta =\frac{t}{t_{{\mathrm{cri},H}_{2}O}}$$

(18) $$\pi_{25}=1-{\left[1+{\left(\frac{X}{\pi_{6}}\right)}^{\pi_{7}}\right]}^{\pi_{8}}-\pi_{9}\cdot e^{-\frac{{\left(X-0.1\right)}^{2}}{0.005}}$$

(19) $$h_{\mathrm{sol}}\left(X,t\right)=A^{\prime }+B^{\prime }\cdot t+C^{\prime }\cdot t^{2}$$

(20) $$A^{\prime }=\sum_{i=0}^{4}a_{i}\cdot {\left(X\cdot 100\right)}^{i}$$

(21) $$B^{\prime }=\sum_{i=0}^{4}b_{i}\cdot {\left(X\cdot 100\right)}^{i}$$

(22) $$C^{\prime }=\sum_{i=0}^{4}c_{i}\cdot {\left(X\cdot 100\right)}^{i}$$

##### Generating Module & Dehumidification Module

The basic structure and components of both the generating and dehumidification modules are shown in Figure 1. Heat and mass transfer occur simultaneously in each module. In much of the prior literature [28], the *ε*-NTU method has usually been adopted to describe simultaneous heat and mass transfer within the structured packing in each module. However, this method is typically used for finite element analysis. In the present study, self-circulation was employed to ensure adequate mass transfer in each module; therefore, an enthalpy–humidity efficiency modeling method was adopted to develop mathematical models for the generator and dehumidifier, in which enthalpy and humidity efficiency were assumed in reference with previous studies [28]. This method quantifies the sufficiency of heat and mass transfer in regeneration and dehumidification processes, using enthalpy and humidity efficiencies, respectively, as defined in Equations (23)–(26). Combined with the inlet states of the solution and moist air, the outlet states of the humid air from the generator and dehumidifier can be determined using this method.

(23) $$\eta_{\omega ,\mathrm{reg}}=\frac{\omega_{\mathrm{out},\mathrm{air}}-\omega_{\mathrm{in},\mathrm{air}}}{\omega_{e,\mathrm{in}}-\omega_{\mathrm{in},\mathrm{air}}}$$

(24) $$\eta_{h,\mathrm{reg}}=\frac{h_{\mathrm{out},\mathrm{air}}-h_{\mathrm{in},\mathrm{air}}}{h_{e,\mathrm{in}}-h_{\mathrm{in},\mathrm{air}}}$$

(25) $$\eta_{\omega ,\mathrm{deh}}=\frac{\omega_{\mathrm{in},\mathrm{air}}-\omega_{\mathrm{out},\mathrm{air}}}{\omega_{\mathrm{in},\mathrm{air}}-\omega_{e,\mathrm{in}}}$$

(26) $$\eta_{h,\mathrm{deh}}=\frac{h_{\mathrm{in},\mathrm{air}}-h_{\mathrm{out},\mathrm{air}}}{h_{\mathrm{in},\mathrm{air}}-h_{e,\mathrm{in}}}$$

After obtaining the moist air outlet states of the dehumidification and regeneration modules, the outlet solution state parameters can be determined by combining the modules’ energy conservation equations, as shown in Equations (27) and (28).

(27) $$Q_{\mathrm{deh}}=\left({\dot{m}}_{\mathrm{sol},\mathrm{dil}}\cdot h_{1s}-{\dot{m}}_{\mathrm{sol},\mathrm{con}}\cdot h_{6s}\right)={\dot{m}}_{a,A5\text{-}A7}\cdot \left(h_{A5}-h_{A7}\right)$$

(28) $$Q_{\mathrm{reg}}=\left({\dot{m}}_{\mathrm{sol},\mathrm{dil}}\cdot h_{3s}-{\dot{m}}_{\mathrm{sol},\mathrm{con}}\cdot h_{4s}\right)={\dot{m}}_{a,A3\text{-}A4}\cdot \left(h_{A4}-h_{A3}\right)$$

Moreover, the changes in solution concentration and mass flow rate after passing through the dehumidification or regeneration module can be determined from Equations (29)–(31).

(29) $${\dot{m}}_{\mathrm{sol},\mathrm{dil}}\cdot X_{\mathrm{sol},\mathrm{dil}}={\dot{m}}_{\mathrm{sol},\mathrm{con}}\cdot X_{\mathrm{sol},\mathrm{con}}$$

(30) $${\dot{m}}_{w,\mathrm{deh}}=\left({\dot{m}}_{\mathrm{sol},\mathrm{dil}}-{\dot{m}}_{\mathrm{sol},\mathrm{con}}\right)={\dot{m}}_{a,\mathrm{deh}}\left(\omega_{A7}-\omega_{A5}\right)$$

(31) $${\dot{m}}_{w,\mathrm{reg}}=\left({\dot{m}}_{\mathrm{sol},\mathrm{dil}}-{\dot{m}}_{\mathrm{sol},\mathrm{con}}\right)={\dot{m}}_{a,\mathrm{reg}}\left(\omega_{A4}-\omega_{A3}\right)$$

##### Regenerator of Solution

The function of the solution recuperator is nearly the same as that of the CO_2_ recuperator. The energy balance equation is given in Equation (26) as follows.

(32) $$Q_{\mathrm{recuperator},\mathrm{sol}}={\dot{m}}_{\mathrm{sol},\mathrm{con}}\cdot \left(h_{4s}-h_{5s}\right)={\dot{m}}_{\mathrm{sol},\mathrm{dil}}\cdot \left(h_{2s}-h_{1s}\right)$$

#### 3.1.3. Governing Equations of Moist Air Paths

Some of the governing equations for moist-air flow have already been described in the sections above. However, the humidity ratio variations under each process have yet to be defined. In the gas-cooler and in the air preheater, the moist-air humidity ratio remains constant, as shown in Equation (33).

(33) $$\mathrm{For}\;\mathrm{the}\;\mathrm{air-heating}\;\mathrm{process:}\;\omega_{a,\mathrm{out}}=\omega_{a,\mathrm{in}}$$

Under the atmospheric pressure of 101.325 kPa, relative humidity is a function of the dry bulb temperature and humidity ratio, as shown in Equation (34). The Engineering Equation Solver program is adopted to derive those correlations.

(34) $$\varphi_{a}=\varphi \left(\omega ,t\right)$$

It can be inferred that when moist air is heated at a constant humidity ratio, the relative humidity decreases, indicating a greater dehumidification capacity.

#### 3.1.4. Performance Metrics of the Coupled System

The coupled system is believed to perform the air-conditioning function through dehumidification (dehumidifier) and cooling (evaporator I). Thus, the performance metrics are defined from two perspectives: one focuses solely on refrigeration performance, and the other on dehumidification performance. As for the former, the overall refrigeration system performance is the same as that of an ordinary refrigeration system, as defined in Equation (35). The evaporator and the dehumidification module are both located in the air-conditioning region, whose heat loads directly affect the overall refrigeration system performance.

(35) $$\mathrm{COP}=\frac{Q_{e,I}+Q_{\mathrm{deh}}}{W}$$

On the other hand, the coupled system is preferred for applications with high dehumidification requirements. Thus, another performance metric aims to provide a specific description of the dehumidification coefficient of performance of the coupled system, as shown in Equation (36). The latent heat of water vaporization is included in the calculation and is assumed to be constant at 20 °C for simplicity.

(36) $${\mathrm{COP}}_{\mathrm{deh}}=\frac{{\dot{m}}_{w,\mathrm{deh}}}{W}\cdot r_{w,20{}^{\circ}C}$$

Given that the primary operating condition targeted by the coupled system in this study is high-humidity conditions, dehumidification capacity is a key focus of this research. Therefore, in evaluating system performance, this study introduces the performance parameter specific dehumidification energy (SDE), measured in kWh/kg, to quantify the energy required to remove 1 kg of moisture, thereby enriching the dimensions of system performance evaluation. The definition of SDE is also shown as Equation (37).

(37) $$\mathrm{SDE}=\frac{W}{{\dot{m}}_{w,\mathrm{deh}}}\cdot 3600$$

#### 3.1.5. Exergy Analysis Model for the Coupled System

In addition to the performance parameters of the coupled system presented above, this study will also conduct an exergy analysis of the coupled system. As a means of in-depth evaluation of thermodynamic performance in complex systems, exergy analysis can provide clear guidance for future system optimization. It can effectively quantify the energy-quality loss due to multiple heat- and mass-transfer processes in the coupled system and clarify the distribution of exergy loss within it. Previous studies have examined exergy analysis methods for some types of liquid desiccant systems [29,30].

For the complex heat and mass transfer processes between the solution and moist air in the dehumidifier and regenerator, the key lies in ensuring consistent dead-state conditions between the solution and moist air. In this case, the solution’s exergy calculation process is decomposed into two parts. The first part is the physical exergy, defined as the exergy of the solution when the restricted dead state (where the solution is at the environmental temperature and pressure) is taken as the reference; it depends on the solution’s and the environment’s temperatures and pressures. The second part is the chemical exergy, defined as the exergy of the solution when the ultimate dead state (in which the solution is infinitely diluted) is taken as the reference; it depends on the solution’s composition. Therefore, based on previous research, the exergy calculation methods for the solution and moist air are given by the following equations, with the parameters defined in the relevant literature [31].

(38) $$ex_{\mathrm{sol}}=ex_{\mathrm{ph}}(T,p)+ex_{\mathrm{ch}}(X)$$

(39) $$ex_{\mathrm{ph}}(T,p)=h(T,p)-h^{*}(T_{0},p_{0})-T_{0}\cdot \left[s\left(T,p\right)-s^{*}(T_{0},p_{0})\right]$$

(40) $$ex_{\mathrm{ch}}(X)=X\cdot \left[\mu_{\mathrm{sol}}^{*}(X)-\mu_{0,\mathrm{sol}}(X_{0})\right]+(1-X)\cdot \left[\mu_{\mathrm{water}}^{*}(X)-\mu_{0,\mathrm{water}}(X_{0})\right]$$

(41) $$\mu_{0}=\underset{X_{0}\to 0}{\lim}\mu \left(T_{0},p_{0},X_{0}\right)$$

(42) $$\begin{array}{l} ex_{a}(T,\omega )=\left(c_{p,a}+\omega \cdot c_{p,v}\right)\cdot T_{0}\cdot (\frac{T}{T_{0}}-1-\ln\frac{T}{T_{0}})\\ +R_{a}\cdot T_{0}\cdot \left[\left(1+1.608\cdot \omega \right)\cdot \ln\frac{1+1.608\cdot \omega_{0}}{1+1.608\cdot \omega_{0}}+1.608\cdot \omega \cdot \ln\frac{\omega }{\omega_{0}}\right] \end{array}$$

Once the exergies of CO_2_, the solution, and moist air are obtained, the exergy loss for each component can be calculated using Equation (43).

(43) $$Ex_{\mathrm{loss}}=Ex_{\mathrm{in}}-Ex_{\mathrm{out}}$$

To evaluate the performance of the coupled system with respect to exergy, the exergy efficiency is employed, as specified in Equation (44). After being conditioned by Evaporator I and the dehumidifier, the indoor air experiences reductions in both temperature and humidity. Thus, for the coupled system, the exergy change associated with this process can be determined using Equation (45). The compression work supplied by the compressor serves as the system input; owing to its high energy grade, it is directly taken as the input exergy.

(44) $$\eta_{ex}=\frac{Ex_{out,useful}}{Ex_{in,total}}$$

(45) $$Ex_{out,useful}={\dot{m}}_{a,A5\text{-}A6}\left(ex_{A5}-ex_{A6}\right)+{\dot{m}}_{a,A5\text{-}A7}\left(ex_{A5}-ex_{A7}\right)$$

Similar to the previously mentioned specific dehumidification energy (SDE), this study focuses on performance evaluation parameters related to the system’s dehumidification capacity. Thus, from an exergy analysis perspective, this study proposes the performance parameter Specific Dehumidification Exergy Loss (SDEL), which characterizes the exergy loss per kilogram of moisture removed as Equation (46). In addition to the system’s overall exergy efficiency and the exergy loss distribution of each component, this study will analyze the variation in the overall energy-quality loss across different operating conditions, as quantified by the SDED.

(46) $$\mathrm{SDEL}=\frac{Ex_{\mathrm{loss},\mathrm{total}}}{{\dot{m}}_{w,\mathrm{deh}}}$$

### 3.2. Assumptions and Working Conditions

#### 3.2.1. Assumptions

Proper assumptions are necessary for theoretical analysis in thermodynamic modeling to clarify the key mechanisms of the coupled system. For the present thermodynamic analysis, assumptions are listed as follows:

- Systematic thermal insultation is perfect, and the exergy losses along pipes and fans are neglected.
- No mass and energy leakage in the system; mass and energy transfer can only occur along specific paths.
- Evaporator I is only considered to cool the air without any latent heat load. For the coupled system designed to meet sensible heat load and latent heat load independently, moist removal is assumed to occur in the dehumidifier only.
- The moist air in the coupled system is considered an ideal gas to simplify the calculations. Its thermal properties and exergies can be calculated through the weighted algorithms of dry air and water vapor.
- The heat exchanger is theoretically modeled using the maximum heat transfer rate–heat exchanger efficiency method. Several key temperature-difference parameters, such as the difference between the compressor discharge temperature and the diluted solution temperature at the solution-heater outlet, are assumed in the calculations.
- The modeling of the dehumidifier and regenerator adopts the method described previously as Equations (23)–(31). By assuming constant enthalpy and humidity efficiency, and combining the enthalpy–humidity efficiency model, thermodynamic modeling is conducted for the dehumidifier and regenerator, where heat and mass transfer occur simultaneously. The values of the enthalpy efficiency and humidity efficiency are assumed based on previous experimental studies. It is worth noting that when modeling the dehumidifier and regenerator using this method, the outlet moist-air state can be determined once the inlet solution state is specified.
- Set the state of the solution under infinite dilution as the dead state for exergy calculation of the solution, to ensure consistency with the dead state of humid air.
- The power of each pump is neglected due to their smaller order of magnitude compared with those energy input components such as compressor.

#### 3.2.2. Working Conditions

In addition, the coupled system thermodynamic model established in this paper must be solved by combining the aforementioned assumptions with reasonable ranges for key variables. The coupled system primarily comprises the CO_2_ transcritical refrigeration cycle, the liquid desiccant dehumidification cycle (i.e., the solution cycle), and the moist-air paths (indoor and outdoor). Table 3 presents the range of input variable values for each cycle or path.

### 3.3. Reliability of the Present Study

Modeling methods of the coupled system in this article is based on each independent parts, including the CO_2_ transcritical refrigeration cycle, aqueous LiCl desiccant dehumidification cycle, and exergy analysis module. For the CTRC, the controlling equations for the compressor, heat exchanger and throttling process, as well as the thermophysical property calculation of CO_2_ based on the NIST REFPROP program, are consistent with the classic experimental research on transcritical CO_2_ systems [32]. For the LiCl liquid desiccant dehumidification cycle, the thermophysical property correlations of the LiCl aqueous solution are derived from the experimental fitting results [26,27], which is widely applied in the field of liquid desiccant dehumidification. The enthalpy–humidity efficiency model for describing heat and mass transfer in the dehumidifier/regenerator has been fully verified by experimental tests [28]. For the exergy analysis module, the method of decomposing the solution exergy into physical and chemical exergy for moist air and LiCl solution is a common way for exergy analysis of liquid desiccant cycles [29,30,31], which have ensured the consistence of humid air and liquid desiccant at dead states. The modeling methods mentioned above are all proven to be reliable according to previous researchers, which also secures the reliability of the results in the present study.

## 4. Results and Discussions

### 4.1. Influence of Working Conditions on the Coupled System Performance

**Figure 8:**
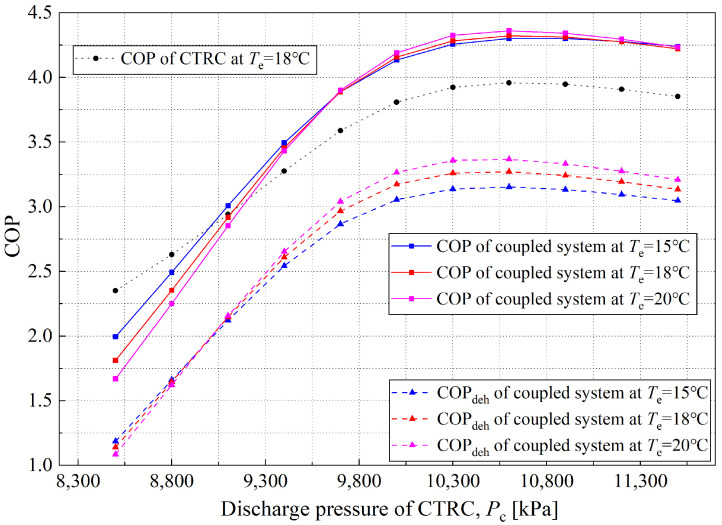
Variation of coupled system COP/COP_deh_ with CTRC discharge pressure under different evaporating temperatures.

From the perspective of system coupling, the exothermic side of the CO_2_ transcritical refrigeration cycle directly serves as a heat source for the solution dehumidification cycle, which concentrates and regenerates the dilute solution, restoring its dehumidification capability. In this coupled system, the discharge pressure of the CO_2_ transcritical refrigeration cycle determines the compressor discharge temperature, which in turn affects the heating and concentration of the dilute solution. As shown in Figure 6a,c, the inlet solution temperature of the regenerator is the key factor that influences the concentration distribution of the outlet solution. Figure 8 shows the variation of the COP and COP_deh_ with discharge pressure at the operating condition where the mass flow ratio of CO_2_ in Evaporator II is 0.7, for different evaporating temperatures.

As shown in Figure 8, both the coefficient of performance (COP) and the dehumidification coefficient of performance (COP_deh_) of the coupled system increase with the compressor discharge pressure at all evaporating temperatures from the beginning, then decrease. Specifically, at an evaporating temperature of 18 °C, when the discharge pressure exceeds 9100 kPa, the overall COP of the coupled system exceeds that of the standalone CTRC, reaching a maximum of 4.32 at 10,600 kPa. According to Equation (35), the numerator of the overall system’s COP primarily comprises the sensible heat load handled by Evaporator I and the psychrometric heat load (both latent and sensible) processed by the dehumidifier. Analysis of the calculation results indicates that as the CTRC discharge pressure increases, the CO_2_ temperature at the compressor outlet (Point 2) rises, thereby substantially increasing the lithium chloride solution concentration. Therefore, at high discharge pressures, the dehumidifier’s contribution to the system COP gradually becomes pronounced. It can be inferred that under low discharge-pressure conditions, the dehumidification capacity of the liquid desiccant cycle is relatively weak because only CO_2_ flows through Evaporator I, which provides refrigeration capacity and contributes to the overall COP. It can also be observed that at low discharge pressures, the dehumidification module’s heat load is low, resulting in a relatively low COP for the coupled system. In contrast, as discharge pressure increases, the rise in discharge temperature indirectly strengthens the mass-transfer driving force and the dehumidification efficiency of the liquid desiccant cycle, thereby improving the overall COP of the coupled system.

However, when the compressor discharge pressure (*P*_c_) is increased to around 10,500 kPa, the COP of the coupled system begins to decline. The calculation results indicate that the core reasons for this phenomenon are as follows: excessively high discharge pressure reduces the COP of the CTRC, thereby reducing the cooling capacity per unit power consumption. On the other hand, the rise in discharge temperature caused by increased discharge pressure results in an excessively high temperature for the dilute solution being regenerated. Due to the constraint of the regenerator’s inherent heat and mass transfer efficiency, the concentration degree of the solution cannot be further improved. Therefore, during this process, the growth rates of the regeneration and dehumidification capacities of the liquid desiccant dehumidification cycle slow, whereas the total power consumption of the coupled system continues to rise steadily. Consequently, the system’s COP first increases and then decreases. This law can be further verified by analyzing trends in the variation of COP_deh_. In summary, from the perspective of the COP index, the coupled system has an optimal discharge pressure. For instance, at an evaporating temperature of 18 °C, the optimal discharge pressure is approximately 10,500 kPa. Additionally, when the discharge pressure reaches a threshold, the COP of the coupled system exceeds that of the standalone CTRC system, demonstrating the coupled system’s performance priority.

In addition to the CO_2_-solution heat exchanger, Evaporator II serves as another key coupling component in the integrated system. Therefore, the ratio *λ* of the CO_2_ mass flow rate in Evaporator II to the total mass flow rate is a key parameter that significantly affects system performance.

**Figure 9:**
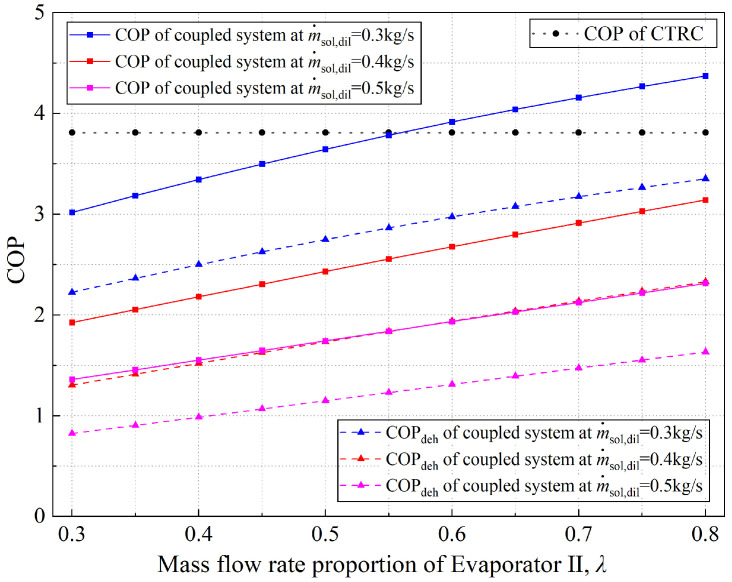
Variation of coupled system COP/COP_deh_ with mass flow rate proportion of Evaporator II under different solution mass flow rate.

Figure 9 illustrates the trends in the COP and COP_deh_ as functions of *λ* across different solution mass flow rates, with the evaporating temperature and discharge pressure fixed at 18 °C and 10,000 kPa, respectively. As shown in the figure, both the COP and COP_deh_ increase monotonically with *λ*; however, compared with the variation amplitude of the COP with discharge pressure, the curves of the COP and COP_deh_ as functions of *λ* are much gentler. From the perspective of the system thermodynamic model, the temperature and concentration of the dilute solution at the dehumidifier inlet are known. Combined with the humidity–efficiency and enthalpy–efficiency models, the variation in regenerator water content can be determined. According to the law of mass conservation, the variation in water content in the solution on the dehumidifier side must be consistent with that on the regenerator side, so the dehumidification capacity of the dehumidifier is clarified in the calculation. When *λ* is small, although the dehumidifier has a specific moisture absorption capacity, the degree of solution cooling is insufficient. To ensure that the dehumidifier’s water content variation matches that of the regenerator, the system dynamically adjusts the solution concentration. For example, when *λ* = 0.3, the dehumidification capacity of the solution on the dehumidifier side is weak, and the system adjusts the concentration of the concentrated solution to 33.32%. When *λ* = 0.8, the concentration of the concentrated solution decreases to 29.96%. This is because the variation in water content during regeneration depends on whether the dilute solution concentration meets the regenerating requirement. In contrast, during dehumidification, it depends on the concentration of the concentrated solution.

In addition, when *λ* is small, the temperature drop range of the concentrated solution is limited. When indoor humid air flows through the dehumidifier, although moisture can be separated, sensible heat generated during heat transfer is returned to the moist air, resulting in a phenomenon in which the humidity of the humid air decreases while its temperature increases. This is also an essential reason for the variation of the COP with *λ*. The variation of the COP and COP_deh_ with the mass flow rate of the diluted solution is obvious in terms of the regenerator’s regenerating capability.

**Figure 10:**
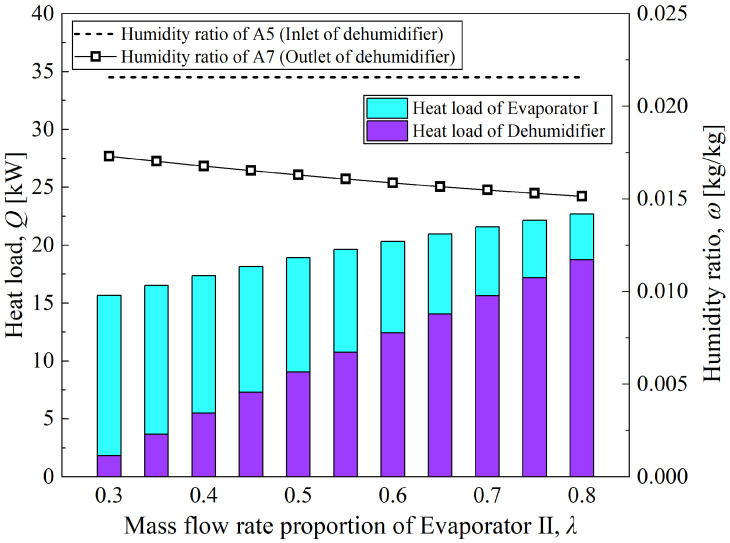
Variation of heat loads and humidity ratios with mass flow rate proportion of Evaporator II.

At a solution mass flow rate of 0.3 kg/s, Figure 10 illustrates the variation in coupled system performance with the mass flow ratio *λ* as a function of two variables: heat load and moisture content. Similar to the variation law in Figure 9, the growth amplitude of the total heat load of the coupled system with *λ* is in accordance with the variation trend of COP. This is because all operating conditions shown in the figure are based on the same CTRC, with compressor power consumption held constant. As *λ* increases, the solution concentration dynamically adjusts in response to the cooling amplitude of the concentrated dehumidification solution, ultimately promoting a gradual rise in the water content variation in the solution in both the regenerator and dehumidifier (*ṁ*_w,deh_ increases from 0.004707 kg/s to 0.007091 kg/s). On the other hand, the sufficient cooling of the concentrated solution in the dehumidifier improves its dehumidification limit for humid air (*ω*_e,6s_ decreases from 0.01089 kg/kg to 0.005483 kg/kg). Driven jointly by the above two factors, the growth rate of the heat load (sum of latent heat and sensible heat) of the dehumidifier in the coupled system exceeds the decline rate of the cooling capacity of Evaporator I, which finally results in the synchronous upward trend of the total system heat load and COP.

**Figure 11:**
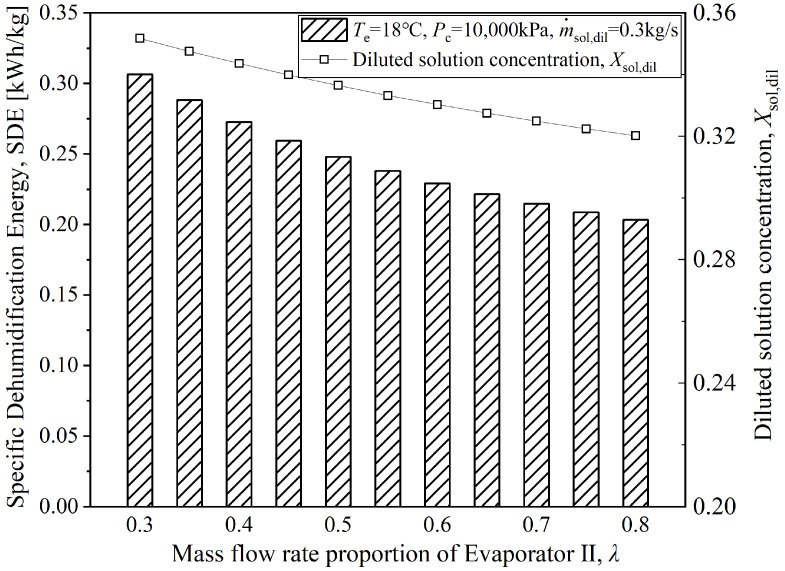
Variation of coupled system *SDE* and with mass flow rate proportion of Evaporator II.

Figure 11 presents the variation relationships between the SDE of the coupled system, the dilute solution concentration, and the mass flow rate proportion of CO_2_ in Evaporator II (denoted as *λ*) under specific operating conditions. The variation in the dilute solution concentration with *λ*, as shown in the figure, is explained in the analysis of the previous figures. Moreover, the figure shows that the energy required to remove 1 kg of moisture by the coupled system under this operating condition ranges from 0.2033 to 0.3063 kWh/kg. Compared with theoretical and experimental results reported in the literature [29,30], the SDE of the coupled system proposed in this paper is much lower, indicating good economic efficiency for dehumidification.

### 4.2. Exergy Performance of the Coupled System

**Figure 12:**
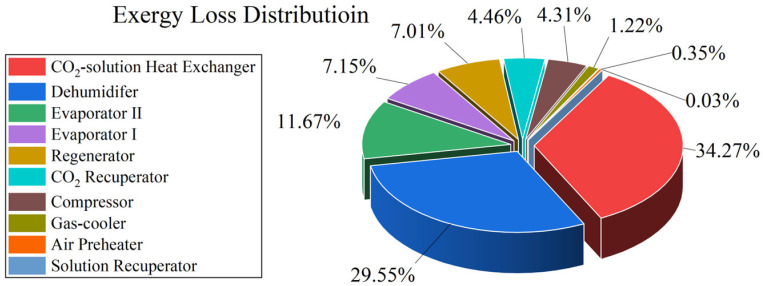
Exergy loss distribution of the coupled system when *T*_e_ = 18 °C, *P*_c_ = 10,000 kPa, *λ* = 0.5, *ṁ*_sol,dil_ = 0.3 kg/s.

Exergy analysis of complex systems aims to evaluate the efficiency of energy utilization, accurately identify the key device at which energy-grade loss occurs, and provide theoretical support for optimal system design. Based on the exergy calculation methods for CO_2_, solution, and moist air described above in this paper, the exergy loss of each component in the system can be analyzed and calculated via Equation (43). Figure 12 shows the distribution characteristics of exergy loss of each component under a specific operating condition.

As shown in Figure 12, exergy loss is primarily due to heat and mass transfer processes within the components, including the CO_2_-solution heat exchanger, regenerator, Evaporator II, Evaporator I, dehumidifier, CO_2_ recuperator, and compressor. Among these components, the exergy losses of the CO_2_-solution heat exchanger and the regenerator account for more than 60% of the system’s total exergy loss. In the following discussion, the exergy losses of the solution recuperator, air preheater, and gas-cooler will be neglected, as they are relatively small compared to those of other devices.

**Figure 13:**
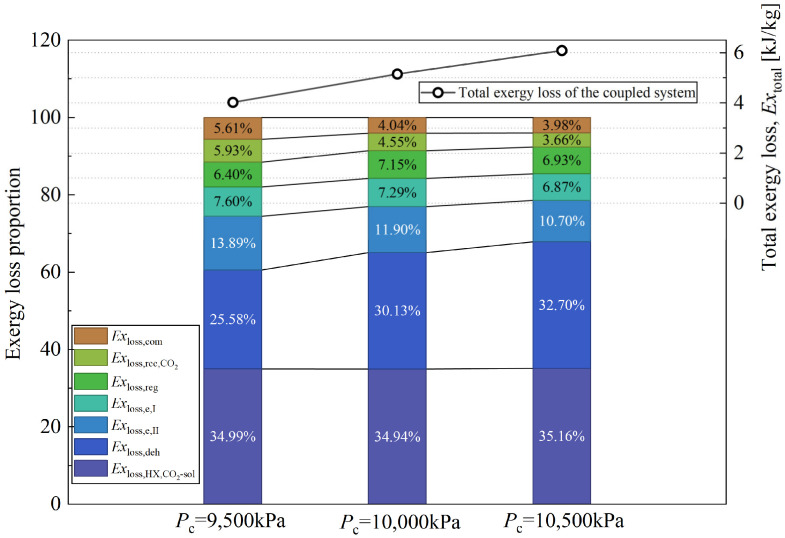
Exergy loss proportion of each device of the coupled system when *T*_e_ = 18 °C, *λ* = 0.5, *ṁ*_sol,dil_ = 0.3 kg/s at different discharge pressure.

Figure 13 illustrates the exergy loss proportion of each principal component and the total exergy loss of the coupled system under different discharge pressure conditions. It can be observed from the figure that the total exergy loss of the system increases with increasing discharge pressure. In terms of the proportion of exergy loss per component, the dehumidifier is the primary contributor to the total exergy loss. As the discharge pressure increases from 9500 kPa to 10,500 kPa, although the system’s total exergy loss increases, the dehumidifier’s exergy loss ratio rises from 25.58% to 32.7%. Combined with the analysis above, it can be inferred that as the discharge pressure increases, the concentration of the dilute solution on the regenerator side increases significantly. This characteristic substantially enhances the mass-transfer driving force on the dehumidifier side (i.e., the difference between the saturated vapor pressure at the surface of the concentrated solution and the partial pressure of water vapor in the moist air). According to existing studies [31], in heat- and mass-transfer components, the primary affections of exergy loss are large temperature and concentration differences (i.e., the mass-transfer driving force). This also explains why the coupled system proposed in this paper exhibits a higher COP and larger total cooling and dehumidification loads when the dilute solution is fully concentrated, while the exergy loss increases accordingly.

**Figure 14:**
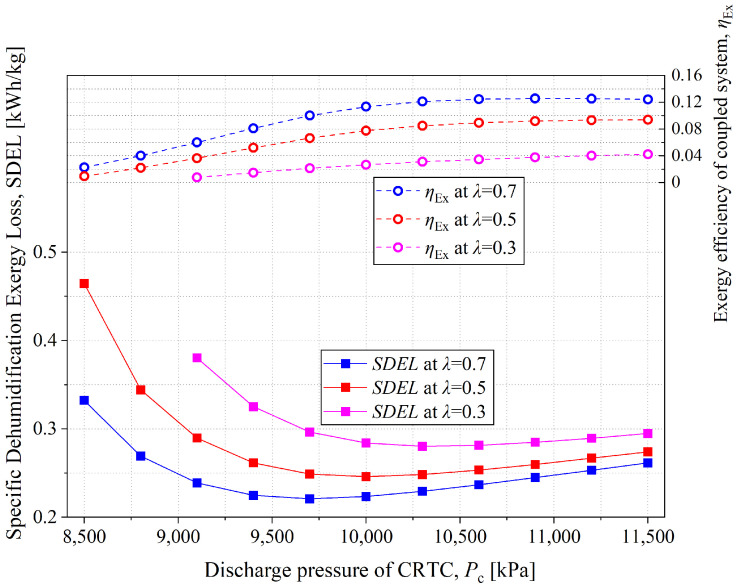
Exergy loss proportion of each device of the coupled system when *T*_e_ = 18 °C, *λ* = 0.5, *ṁ*_sol,dil_ = 0.3 kg/s.

Figure 14 presents the variation laws of the Specific Dehumidification Exergy Loss (SDEL, i.e., the exergy loss required to remove per kilogram of moisture) and the exergy efficiency of the coupled system with discharge pressure under different *λ* conditions. As shown in the figure, SDEL first decreases and then increases with increasing discharge pressure. Combined with the definition formula of SDEL (Equation (46)) and the calculation results, it can be inferred that in the range where SDEL decreases with the increase in discharge pressure, the core reason is that the concentration degree of the concentrated solution used for dehumidification gradually becomes sufficient. As the discharge pressure increases, the corresponding discharge temperature rises, thereby increasing the heating capacity of the dilute solution. As a result, the dehumidification capacity of the coupled system is significantly enhanced. As the mass-transfer driving force increases, the numerator (total exergy loss) in Equation (46) decreases, reducing SDEL consequently. The system’s exergy utilization efficiency is optimal. However, with a further increase in discharge pressure, the solution becomes overconcentrated, resulting in an excessively strong mass-transfer driving force in the dehumidifier. At this point, the exergy loss of the dehumidifier gradually increases and becomes dominant, which is the key reason for the upward trend of SDEL in the later stage of increasing discharge pressure. The influence of *λ* on SDEL can also be analyzed based on the core logic of “whether the mass transfer driving force is enormous under the premise of ensuring dehumidification capacity”, which will not be elaborated here. In summary, from the perspective of exergy utilization efficiency for moisture removal, the coupled system has an optimal operating state—it can not only ensure that dehumidification capacity meets requirements but also avoid excessive energy-quality loss caused by an overly large mass-transfer driving force.

The exergy efficiency of the coupled system ranges approximately from 0.2% to 12.4%. As shown in Figure 14, the exergy efficiency increases gradually and then levels off. Analysis of the calculation results shows that as the discharge pressure increases slowly, the system’s energy consumption increases accordingly. However, the deviation from the low-temperature, low-humidity environmental reference state in the air output by the dehumidifier becomes more significant, leading to a corresponding increase in the system’s useful exergy. The two grow at similar rates, and the cooling capacity of Evaporator I increases with rising discharge pressure (this law can be verified by the variation in COP in CTRC shown in Figure 8). Eventually, the air temperature drop at the outlet of Evaporator I is also quite considerable. Under the combined effect of these two factors, the exergy efficiency exhibits a noticeable upward trend during the early stage of the discharge pressure increase. The flat trend of exergy efficiency in the later stage of the increase in discharge pressure can also be explained by the COP variation law of CTRC in Figure 8: with the further increase in discharge pressure, the exergy change in the moist air generated by the dehumidifier and the compressor power consumption increase synchronously, but the cooling capacity of Evaporator I no longer increases, corresponding to the downward trend of CTRC’s COP in Figure 8, which ultimately leads to the stabilization of exergy efficiency.

From the exergy efficiency results, exergy efficiency is lower than regular coupled system with traditional heat pump and liquid desiccant dehumidification system [30]. In the study of Qinling Zhang, a heat-pump-driven liquid desiccant dehumidification system is proposed, with exergy efficiency of about 20.1%. Exergy losses of the proposed coupled system mainly occur in the regenerator. Compared with the normal vapor compression refrigeration cycle, CTRC owns a high discharge temperature which leads to huge exergy losses due to the temperature difference between supercritical CO_2_ and diluted solution. But the solution can be concentrated much further in the regenerator, causing great dehumidifying potential. However, the highly concentrated solution will need more cooling for dehumidifying which also leads to exergy losses. Thus, the coupled system is considered to meet deep-dehumidification situations, whose cost is less exergy efficiency. Accordingly, the results indicate that the exergy efficiency leaves room for optimization in the coupled system, such as multi-stage configurations.

## 5. Conclusions

This paper proposes an air conditioning system that achieves deep coupling between the CO_2_ transcritical refrigeration cycle (CTRC) and the liquid desiccant dehumidification cycle. Theoretical analysis of the system is conducted through modeling combined with an exergy analysis model. Based on sufficient analysis and discussion, the following conclusions regarding this coupled system are drawn:

(1) The coefficient of performance (COP) and dehumidification coefficient of performance (COP_deh_) of the proposed coupled system integrating the CO_2_ transcritical refrigeration cycle (CTRC) and liquid desiccant dehumidification cycle first increase and then decrease with the discharge pressure of CTRC, featuring an optimal discharge pressure. Otherwise, under some specific operating conditions, the system’s COP can surpass that of the standalone CTRC system, demonstrating excellent comprehensive refrigeration and dehumidification performance.
(2) Key parameters such as the mass flow rate proportion *λ* of CO_2_ in Evaporator II and the mass flow rate of the dilute solution exert a significant impact on system performance. Appropriately increasing *λ* can improve the COP and dehumidification capacity, and the system exhibits low specific dehumidification energy (SDE), indicating outstanding dehumidification economy.
(3) The exergy loss of the system is mainly concentrated in the CO_2_-solution heat exchanger and the regenerator (accounting for over 60% of the total exergy loss under specific operating conditions). The system can be adjusted to an optimal operating state that balances dehumidification capacity and exergy loss.
(4) Owing to the features of CTRC, the coupled system’s exergy efficiency is below 12.4% due to large exergy loss within processes such as high-temperature-difference heat transfer. Therefore, there still exists room for system optimization in the future research, such as a stepwise energy utilization method. Moreover, experimental research is necessary to proceed, helping to validate and optimize the coupled system.

## Figures and Tables

**Table 1:** Coefficients of Equations (13)–(18).

**Coefficient**	***π*_0_**	***π*_1_**	***π*_2_**	***π*_3_**	***π*_4_**
Value	0.28	4.30	0.60	0.21	5.10
**Coefficient**	***π*_5_**	***π*_6_**	***π*_7_**	***π*_8_**	***π*_9_**
Value	0.49	0.362	−4.75	−0.40	0.03

**Table 2:** Coefficients of Equations (19)–(22).

**Coefficient**	***a*_0_**	***a*_1_**	***a*_2_**	***a*_3_**	***a*_4_**
Value	−66.2324	11.2711	−0.79853	21,534·10^−2^	−1.66352·10^−4^
**Coefficient**	***b*_0_**	***b*_1_**	***b*_2_**	***b*_3_**	***b*_4_**
Value	4.5751	−0.146924	6.30723·10^−3^	−1.38054·10^−4^	1.0669·10^−6^
**Coefficient**	***c*_0_**	***c*_1_**	***c*_2_**	***c*_3_**	***c*_4_**
Value	−8.09689·10^−4^	2.18145·10^−4^	−1.36194·10^−5^	3.20998·10^−7^	−2.64266·10^−9^

**Table 3:** Key input variables of the thermodynamic model with their values and ranges.

Cycles	Variables	Units	Values or Ranges
CO_2_ transcritical refrigeration cycle	Evaporating temperature, *T*_e_	°C	15–20
	Discharge pressure, *P*_c_	kPa	8500–11,500
	Superheated degree, Δ*T*_sup_	K	5
	Temperature at outlet of gas-cooler, *T*_5_	°C	*T*_A1_ + 5
	CO_2_ recuperator efficiency, *η*_recuperator,CO_2__	%	0.8
	CO_2_-solution heat exchanger efficiency, *η*_HX,CO_2_-sol_	%	0.8
	Isentropic efficiency of compressor, *η*_isen_	%	0.9
	Mass flow rate proportion in evaporator II, *λ*	%	0.2–0.9
Solution dehumidification Cycle	Diluted solution mass fraction of LiCl, *X*_sol,dil_	%	0.25–0.45
	Solution recuperator, *η*_recuperator,sol_	%	0.8
	Humidity efficiency of the dehumidification module, *η_ω_*_,deh_	%	0.5
	Enthalpy efficiency of the dehumidification module, *η_h_*_,deh_	%	0.4
	Humidity efficiency of the regenerating module, *η_ω_*_,reg_	%	0.5
	Enthalpy efficiency of the regenerating module, *η_h_*_,reg_	%	0.4
Moist-air path (outdoor)	Moist air inlet temperature, *T*_A1_	°C	35
	Moist air inlet relative humidity, *φ*_A1_	%	0.85
Moist-air path (indoor)	Moist air inlet temperature, *T*_A5_	°C	30
	Moist air inlet relative humidity, *φ*_A5_	%	0.8

## Data Availability

The original contributions presented in this study are included in the article. Further inquiries can be directed to the corresponding author.

## Nomenclature

COP	Coefficient of performance
*ex*	Specific exergy, kJ/kg
*Ex*	Exergy, kJ
*h*	Specific enthalpy, kJ/kg
*ṁ*	Mass flow rate, kg/s
*P*	Absolute pressure, kPa
*Q*	Heat load, kW
*R*	Gas constant, kJ/(kg·K)
*r*	Latent heat of vaporization, kJ/kg
*s*	Specific entropy, kJ/(kg·K)
*SDE*	Specific dehumidification energy, kWh/kg
*SDEL*	Specific dehumidification exergy loss, kWh/kg
*t*	Temperature, °C
*T*	Temperature, K
*V*	Volume flow rate, m^3^/s
*v*	Specific volume, m^3^/kg
*W*	Power, kW
*X*	Concentration of solution
Greek symbols	
*η*	Efficiency
*λ*	mass flow rate ratio in Evaporator II
*μ*	Chemical potential, kJ/kg
*φ*	Relative humidity
*ω*	Humidity ratio of moist air, kg/kg
Subscripts	
a	Moist air
ch	Chemical
CO_2_	Carbon dioxide
con	Concentrated
deh	Dehumidification/dehumidifier
dil	Diluted
e	Equilibrium state with solution
HX	Heat exchanger
isen	Isentropic process
ph	Physical
r	Refrigerant
reg	Regenerator
sat	Saturated state
sol	Solution
v	Volume
w	Water
I	Evaporator I
II	Evaporator II
0	Dead state
